# HPV vaccine decision making in pediatric primary care: a semi-structured interview study

**DOI:** 10.1186/1471-2431-11-74

**Published:** 2011-08-30

**Authors:** Cayce C Hughes, Amanda L Jones, Kristen A Feemster, Alexander G Fiks

**Affiliations:** 1 Center for Pediatric Clinical Effectiveness and Policy Lab, The Children's Hospital of Philadelphia, 3535 Market Suite, Philadelphia, PA, 19104, USA; 2 Department of Pediatrics, The Children's Hospital of Philadelphia, 3535 Market Suite, Philadelphia, PA, 19104, USA; 3 Department of Infections Diseases, The Children's Hospital of Philadelphia, 3535 Market Suite, Philadelphia, PA, 19104, USA; 4 Leonard Davis Institute of Health Economics, The University of Pennsylvania School of Medicine, Colonial Penn Center, 3641 Locust Walk, Philadelphia, PA 19104, USA; 5 Pediatric Research Consortium, The Children's Hospital of Philadelphia, 3535 Market Suite, Philadelphia, PA, 19104, USA

## Abstract

**Background:**

Despite national recommendations, as of 2009 human papillomavirus (HPV) vaccination rates were low with < 30% of adolescent girls fully vaccinated. Research on barriers to vaccination has focused separately on parents, adolescents, or clinicians and not on the decision making process among all participants at the point of care. By incorporating three distinct perspectives, we sought to generate hypotheses to inform interventions to increase vaccine receipt.

**Methods:**

Between March and June, 2010, we conducted qualitative interviews with 20 adolescent-mother-clinician triads (60 individual interviews) directly after a preventive visit with the initial HPV vaccine due. Interviews followed a guide based on published HPV literature, involved 9 practices, and continued until saturation of the primary themes was achieved. Purposive sampling balanced adolescent ages and practice type (urban resident teaching versus non-teaching). Using a modified grounded theory approach, we analyzed data with NVivo8 software both within and across triads to generate primary themes.

**Results:**

The study population was comprised of 20 mothers (12 Black, 9 < high school diploma), 20 adolescents (ten 11-12 years old), and 20 clinicians (16 female). Nine adolescents received the HPV vaccine at the visit, eight of whom were African American. Among the 11 not vaccinated, all either concurrently received or were already up-to-date on Tdap and MCV4. We did not observe systematic patterns of vaccine acceptance or refusal based on adolescent age or years of clinician experience. We identified 3 themes: (1) Parents delayed, rather than refused vaccination, and when they expressed reluctance, clinicians were hesitant to engage them in discussion. (2) Clinicians used one of two strategies to present the HPV vaccine, either presenting it as a routine vaccine with no additional information or presenting it as optional and highlighting risks and benefits. (3) Teens considered themselves passive participants in decision making, even when parents and clinicians reported including them in the process.

**Conclusions:**

Programs to improve HPV vaccine delivery in primary care should focus on promoting effective parent-clinician communication. Research is needed to evaluate strategies to help clinicians engage reluctant parents and passive teens in discussion and measure the impact of distinct clinician decision making approaches on HPV vaccine delivery.

## Background

Since 2006, vaccines have been licensed to prevent cervical cancer, genital cancers, precancerous lesions, genital warts, and other conditions caused by human papillomavirus (HPV) [1-3]. As a preventive intervention, the HPV vaccine is especially important since HPV is the most common sexually transmitted infection [4] and is often acquired shortly following sexual initiation [5]. The population at risk for acquiring HPV includes nearly half of all US high school students, [6] and an estimated 33.8% of 14 to 24 year olds are currently infected [4].

Multiple clinical trials have demonstrated the safety and efficacy of the HPV vaccine,[7,8] and national guidelines recommend the HPV vaccine for girls 11-12 years of age as part of the routine adolescent vaccine platform [9]. However, despite these recommendations, rates of HPV vaccination remain the lowest of all adolescent vaccines according to the 2009 National Immunization Survey [10,11]. As of 2009, only 27% of US girls between 13 and 17 years of age are fully vaccinated. Although the HPV vaccine is relatively new, this finding is especially concerning since full protection requires three doses given over a minimum of six months [9].

In an effort to increase vaccination rates, numerous studies have examined factors associated with the acceptability of HPV vaccine among parents and adolescents, and factors associated with clinician intention to vaccinate, both of which were high [12,13]. Importantly, parents, teens, and clinicians all report that their views on acceptability are influenced by each other. Effective communication about HPV among parents, teens and providers has been linked to vaccine acceptability and receipt [14,15]. Parents and adolescents are positively influenced by getting a doctor's recommendation,[16-18] adolescents stress the importance of parents, partners and other significant people supporting vaccination, especially mothers,[15,19-21] and clinicians' intention to vaccinate depends in part on their perception of parents' concerns regarding safety and efficacy [22-24]. Clinicians are also affected by normative influences such as national guidelines and recommendations [24,25].

Prompted by the discrepancy between high reported vaccine acceptability and intention to vaccinate and low vaccination rates, we conducted a qualitative study using individual interviews with mother, adolescent, and primary care clinician triads to better understand decision making at the point of care. Discussion about barriers to vaccine receipt has generally focused separately on parents, clinicians or adolescents. We studied triads to move beyond conceptualizations of barriers among individuals, and to instead examine the interaction at the visit. By incorporating three distinct perspectives and focusing on decision making in the primary care setting, we sought to generate hypotheses to inform interventions to increase vaccine receipt.

## Methods

This qualitative study was conducted within The Children's Hospital of Philadelphia (CHOP) Pediatric Research Consortium (PeRC), a multi-state, hospital-owned, primary care practice-based research network including more than 235,000 children and adolescents. Study practices included 4 urban teaching practices where fewer than 35% of patients have private insurance and 5 practices not involved in resident teaching, where over 80% of children are privately insured. The study was conducted in a health system where the HPV vaccine was covered by insurance, which allowed us to look at factors other than cost that might impact the decision-making process.

### Study design and patient population

We conducted semi-structured individual interviews with 20 mother-adolescent-clinician triads. Based on the emphasis in the existing literature on the impact of maternal input on HPV vaccine decision making, we excluded male caregivers [14,15,18,26]. We purposively sampled to achieve a balance of adolescent ages, (11-18) and a balance of practice settings, and conducted 60 interviews between March and June 2010. No more than three triads were chosen from each participating practice. We sampled only attending physicians, none of whom had specific training in adolescent medicine. Six triads declined; 5 due to parental or clinician time constraints and 1 due to an adolescent's medical complexity.

Rosters of eligible patients were generated from CHOP's electronic health record and included all girls ages 11-18 that had not initiated the HPV vaccine series and also had an upcoming well-visit at one of the participating sites. We chose to focus on preventive well-visits, as the American Academy of Pediatrics (AAP) recommends that immunizations be administered by primary care clinicians during these routine preventive visits and the HPV vaccine is typically discussed between US families and clinicians in this setting [27]. After a visit at which the adolescent had an opportunity to receive the HPV vaccine, we approached the mother, adolescent, and clinician to introduce the study and obtain consent/assent. Interviews were conducted immediately after the visit, in a private room at the practice, and each member of the triad was interviewed separately. Interviews were conducted by either a pediatric resident (ALJ) or a non-clinical research assistant with experience in conducting qualitative interviews (CCH). Themes were consistent across interviews. This study was approved by the CHOP Institutional Review Board, an official recognized research ethics committee.

### Data collection

Through a detailed review of the relevant literature, meetings of the research team, and consultation with outside experts, we developed an interview guide to elicit parent, adolescent and clinician perspectives on the decision to recommend (clinician) and receive/refuse (mothers and adolescents) adolescent vaccines (guide available upon request). Parents and adolescents were asked about their prior knowledge of vaccines, whether they received vaccines at the visit, whether vaccines were discussed with the clinician, and how they made the final decision to either receive or refuse available vaccine(s). We asked clinicians to focus on the specific visit and describe the vaccine discussion, compare their presentation of HPV with other vaccines, and describe their perception of the family's decision. Two trained interviewers (AJS and CCH) conducted the interviews. Themes were consistent across both interviewers. Using a brief questionnaire, we collected demographic data on participants.

### Data analysis

Interviews were audio taped and analyzed using NVivo qualitative data analysis software (QSR International, Melbourne, Australia). Analysis was based on a modified grounded theory approach, in which coding was inductive and not based on an a priori coding framework [28]. To take advantage of our study design, we analyzed data both within and across triads. Data collection was concurrent with data analysis, and continued until we reached saturation, with no new themes emerging. Two authors (ALJ and CCH) independently coded the interviews. Using an iterative process, we reviewed codes, identified emerging themes, and resolved any discrepancies through consensus. Using NVivo, each transcript was linked with participants' demographic data. After all transcripts were coded, themes were finalized based on the consensus of the research team. Representative verbatim comments were selected for presentation.

## Results

Nine adolescents received the HPV vaccine at the visit and 11 adolescents did not. All 11 that did not receive the HPV vaccine either received other routine adolescent vaccines at their visit or were already up to date, illustrating that HPV was the only vaccine refused. Demographic characteristics of participants are described in Table 1. We did not observe systematic patterns of vaccine acceptance or refusal based on adolescent age or years of clinician experience. Eight of the nine teens who accepted the HPV vaccine were African American. We identified three primary themes highlighted below. Verbatim comments to support these themes are presented in the text and in Table 2.

**Table 1:** Characteristics of study participants

	n
**Mothers**	

Race	
Black	12
White	8
Education	
≤ High School Diploma	9
Some College	1
Bachelor's Degree	5
Master's Degree or Professional Degree	5
Occupation	
Unemployed	5
Working Without Pay	2
Self-Employed	1
Working for Wages	12
Total	20

**Adolescent girls**	

Age	
11-12	10
13-15	7
16-18	3
Received HPV Vaccine*	
Yes	9
No	11
Total	20

**Clinicians**	

Race	
Black	2
White	15
Other	3
Gender	
Female	16
Male	4
Practice Setting	
Urban Resident Teaching Practice	10
Other Practices ^	10
Years in Practice (Post-Training)	
< 10 years	3
10 - 20 years	10
> 20 years	7
Total	20

* Insurance covered HPV vaccine for all subjects.

^ Practices located in either an urban or suburban setting with primarily privately

insured patients and no residents.

**Table 2:** Themes and representative verbatim comments

Theme Comment
**1. Parents delayed, rather than refused vaccination, and when they expressed reluctance, clinicians were hesitant to engage them in discussion**.

***Reasons for delay: children not at risk***
"I don't think she need [HPV vaccine] at this time. I guess it's for girls that's having...sex...and I know my daughter and she's not into that right now." *Mother, age 44*
"I think I would wait until she's a little bit older, 15 or 16. I just don't think they need it right now, at 13...it's young." *Mother, age 48*
"Hopefully [Name] ain't having no sex...but you never know... Hopefully they won't be [sexually active] until they get good and grown. That's what we want but it might not happen." *Mother, age 51*
"I don't think my children are at risk where they need to have it." *Mother, age 47*
"[The HPV vaccine] is appropriate for when girls start dating, and things along those lines." *Mother, age 45*
"I think there's some parents who think 'It's not my kid that's going to be having sex, so they don't need this at all.'" *Clinician, non -teaching practice*
"[Name] is a perfect example...of a girl that you're pretty confident that at 14 is not sexually active...so you have a little bit of leeway." *Clinician, non-teaching practice*
***Reasons for delay: concerns about safety and efficacy***
"I probably just want to know more about it and what the risks are...It's new to me...so I need more education on it." *Mother, age 51*
"I'd like to know the side effects of it...how long they've been testing it." *Mother, age 30*
"I still want to do a little more research...I've heard some pros and cons about it, and since we do have a longer time-line...that's why we're not doing it today." *Mother, age 43*
"[Parents] had concerns about how long the vaccine's been out and the safety, and they wanted to have more time to think about it." *Clinician, urban teaching practice*

***Clinician hesitancy***
"I don't push it the same way as... [other vaccines]." *Clinician, urban teaching practice*
"I will say that I only push it so far...if I had a two-month old who is refusing...IPV or something like that, I might talk to them...I might take up much more time trying to convince them to do that, because I think that the nature of that illness is such that, you know there are devastating consequences...Now, HPV, the truth is that a significant portion of young women will get HPV, but a very large percentage of them will also clear HPV and not actually go on to have cervical cancer. So it's one of those vaccines where I think it's a good idea, but...I don't really twist their arm or anything." *Clinician, urban teaching practice*
"She said she'd prefer to wait and get it later...*Did she give any specific concerns about why she wants to wait *...No, and I didn't push her on that." *Clinician, non-teaching practice*
"We probably don't press it as strong as we do with the other [teen vaccines]." *Clinician, non-teaching practice*
"I'm comfortable with them waiting on the HPV vaccine; I'm not comfortable with them waiting on DTaP or meningitis." *Clinician, non-teaching practice*

**2. Clinicians used one of two distinct strategies to present the HPV vaccine. Neither elicited negative responses from families**.

***Presenting HPV in the same was as other routine vaccines***
"I mention it at 11 because that's when the other booster vaccines are due so that helps me in my endeavor to try to vaccinate, by saying you're due for these vaccines." *Clinician, urban teaching practice*
"I told her about the vaccines she was due for [including HPV]." *Clinician, urban teaching practice*
***Presenting HPV as optional and highlighting the risks and benefits of the vaccine***
"He said [HPV] was something that was optional or we could talk about it later, and the others... were not optional." *Mother, age 39*
"[The doctor] discussed the side effects....and reducing the chances of her getting the virus...that was helpful." *Mother, age 30*
"I do explain that it is optional because...it's not like you have to get it for school or anything like that." *Clinician, urban teaching practice*
"Part of [why we don't press the vaccine] is, at least currently, [because of the] school regulations... the parents will get messages about [the other vaccines] but they won't get messages about [HPV]." *Clinician, non-teaching practice*

**3. Teens considered themselves passive participants in decision making, even when parents and clinicians reported including them in the process**.

*What did you think about not getting the vaccine?* "It doesn't matter, I didn't really care about it either way." *Teen, age 13*
"I think there was one [vaccine I was eligible for] but I'm not sure what it was for exactly, like what it was called." *Teen, age 13*
"I don't like needles...why it got to be a needle? Instead of that shot up the nose like the flu." *Teen, age 12*
*Was your daughter involved in the conversation?* "[My daughter] was here, but of course she didn't want to get it...because she don't like no needles, but I said OK." *Mother, age 51*
"[The decision] was mine. She don't know what [the vaccine] is." *Mother, age 44*

### Parents delayed, rather than refused vaccination, and when they expressed reluctance, clinicians were hesitant to engage them in discussion

Only one mother in our sample was resolute in her decision never to vaccinate her daughter against HPV. Others who declined the vaccine at the visit planned to delay or "wait a year" and revisit the vaccine decision, as opposed to refusing the vaccine outright. One mother said, "We're going to wait on [Gardasil]...she's not even in high school yet...we're just going to wait and see...maybe when she's 16 or 17." When clinicians encountered hesitation from parents, they tended not to "push," instead affirming parents' preference to wait. For example, this family's clinician reported, "I said it's up to them...they can get it now or they can wait." Another clinician, describing her response to a reluctant family, said, "I've learned with them to just back off, go on with the rest of the visit, and we'll address it again next year."

Clinicians anticipated resistance from parents regarding the HPV vaccine, particularly because of its perceived association with sexual activity. One clinician whose patient declined the HPV vaccine at the visit noted, "HPV has so many other implications for parents...it's one they fight you on...because you're suggesting that their child is or will be sexually active soon, and they don't want to hear that." Most parents in our sample did associate the need for HPV vaccine with current sexual activity. For example, one mother explained, "I don't think I'm ready for her to get [the HPV vaccine]...because she's a good kid and I know she ain't out there sexually...I'm just not worried about that right now." Clinicians expressed difficulty in communicating to parents why the vaccine was recommended to girls who were not yet sexually active. One clinician, after discussing the vaccine with a mother who declined the vaccine because she felt her daughter was not at risk, said, "I think people really misunderstand how ineffective the vaccine is if you give it after they start having sex... [We should think about] how to talk to families about that."

Some clinicians shared parents' view that certain teens were not at risk, and could therefore delay vaccination, which may have influenced their decision not to pursue further discussion. This perception was often based on the characteristics of a child or family's demographic characteristics, such as socio-economic status. One clinician whose patient had delayed the vaccine said, "She hasn't even started puberty yet...it's a very close-knit family, the father's a physician, they live in the suburbs... there's no concern about her being sexually active anytime soon." The mother in this triad recalled, "[The doctor] said [HPV] was something that was optional." Another clinician, whose patient received the vaccine, explained, "The family is a more urban family that has experienced some of the urban realities a little more than some of my suburban families."

Clinicians were also sensitive to parents' worries about the safety and efficacy of the HPV vaccine, especially in the long-term. One clinician explained, "A lot of families have seen advertisements about [the vaccine] and have a lot of concerns." Some parents cited these concerns as a reason for delaying vaccination. One mother said, "Based on the clinical studies I've seen, there's not enough long-term data for immunity for me... I don't believe that the efficacy is there." Her 18 year old daughter explained why she did not receive the vaccine, saying, "I guess my mom...wants to wait until [there is] more research on it." Another mother added, "I know it's a fairly new vaccine, so I kind of wanted to give it a few years out there...I'm worried about, as these girls get older, if it will affect their reproductive organs."

Some clinicians did not push reluctant families to accept the HPV vaccine because clinicians perceived it was less important than other vaccines. For example, one clinician in a resident teaching setting explained, "Flu would be the example where people very frequently don't want to get it, and I... say I think they're making a very bad decision, that they're putting their child's life at risk...whereas I can't really say that about HPV." The mother in this triad shared this perception, accepting other vaccines at the visit but not HPV.

### Clinicians used one of two distinct strategies to present the HPV vaccine. Neither elicited negative responses from families

Clinicians used two distinct strategies for presenting the HPV vaccine to families. The first was to recommend the HPV vaccine in the same way as other adolescent vaccines, without providing additional details. One clinician in a resident teaching practice whose patient received the vaccine, explained, "I present [HPV] like it's a routine vaccine...I treat it like any other vaccine." Clinicians who used this approach felt that they had more success than those that distinguished the HPV vaccine as unique. In our sample, four of the six patients whose clinicians used this approach received the HPV vaccine at the visit. One clinician said, "Usually the way I present it, they get all the vaccines. If they don't get all the vaccines, it's because they don't want any of them." The mother in this triad accepted the HPV vaccine, and said she was satisfied with the vaccine discussion.

The second approach was to frame the HPV vaccine as "optional" and highlight the risks and benefits of the vaccine. Five of the fourteen teens whose clinicians used this approach received the HPV vaccine at the visit. A clinician described, "With the required vaccines I say these are shots she's due for today...and then when I talk about HPV, I say she's also eligible to get the HPV vaccine today, but it's your choice." Another clinician said, "I say it's up to them, that they can get it now or they can wait." Some clinicians who used this strategy focused on parents' concerns about the vaccine being "new." For example, one clinician said, "We spend more time... detailing [why we are recommending it] because it's newer." Others noted that, unlike other routine adolescent vaccines, the HPV vaccine was not required for school attendance, so they presented it separately. Mothers in triads where clinicians used this strategy appreciated the additional information clinicians provided. One mother noted, "When he explained what...[the HPV vaccine] was used for and what it helps prevent...I was comfortable with that."

Clinicians had differing beliefs about how much information they should provide families about the HPV vaccine. Those who used the first approach were aware of the absence of a school mandate, but did not share this with parents. One clinician explained, "I don't really say what's required by school and what's not, and I've never had anyone ask me that." In contrast, a clinician that employed the second strategy said, "It's not a required vaccine so I do educate families that it's a recommended vaccine...I think families have a right to know that as part of their decision-making...I don't feel right in presenting something to a family as non-optional for school when that in fact is not true." In both groups, many clinicians described their approach as consistent across visits. In none of the 20 triads in this sample did parents or adolescents complain about the way the vaccine was presented.

### Teens considered themselves passive participants in decision making, even when parents and clinicians reported including them in the process

Clinician, parent, and adolescent interviews consistently revealed that teens played a minimal role in vaccine decision making. Discussion about vaccines was directed primarily to the mother. Even when clinicians reported engaging both adolescents and parents in discussion about the vaccine, mothers were the primary decision-makers. One 11 year old teen, when asked which vaccines she received, said, "Yes, it's the one that my Mom said but I have no clue what it is. I've never heard of it." Even a 16 year old who was seen alone as part of the visit did not want to discuss the vaccine without her mother present. All teens in our sample reported that they were satisfied with their role in the decision making, and parents were also satisfied with the level of teen involvement. When asked if she would prefer to have participated more in the discussion, one 11 year old who received the vaccine replied, "It doesn't matter. I could talk or not." One mother explained, "She didn't really have a choice, it's my decision as a parent."

The main way that adolescents reported taking part in the vaccine discussion was to express concern about anticipated pain from shots. A 14 year old participant whose mother declined the vaccine noted that she had heard the HPV vaccine was more painful than other vaccines; however, even among participants who had never heard of the HPV vaccine, pain was a primary concern. One 11 year old who received the vaccine, when asked if she would have liked information about vaccines prior to the visit, said, "No, I just don't want to get it. I don't like shots. They hurt." Five teens who expressed concern about pain received the vaccine, and four did not, suggesting that pain may not have impacted vaccine receipt in our sample. While some teens hinted at the connection between maturity and the vaccine, in contrast to their parents, none of the adolescents interviewed mentioned sex in discussing the vaccine (Table 2).

## Discussion

We conducted this qualitative study with mothers, adolescents, and clinicians to better understand discrepancies between research findings that show high HPV vaccine acceptability among parents and adolescents, and high clinician intention to recommend the vaccine, but low rates of receipt. By considering the perspectives within and across each triad interviewed, we identified several themes that may help explain low vaccination rates and inform future interventions.

Our study identified that parents tended to delay, rather than refuse vaccination, and that when parents resist vaccination, clinicians often do not directly address their concerns, contributing to the postponement of vaccination. We found several explanations for this pattern. Confirming results from prior studies, parent concerns about vaccine safety and efficacy were reasons parents chose to delay [18,26,29]. However, we found that concerns related to sex were also salient. Parents' perception that teens were not at risk because they were not thought to be currently sexually active often prompted the decision to decline the vaccine and "wait a year" before revisiting the decision. This pattern is concerning since rates of attendance at preventive visits decline after age 11 years and 3 doses are needed for full protection [9,30]. These delays may also be prolonged since parents are known to underestimate their children's sexual activity [31,32]. Further research using a longitudinal design would be useful to determine the length of delays and to identify factors that influence when and how vaccine delay turns into vaccine refusal.

Some clinicians, like parents, believed that certain children, especially those in the target age group for vaccination, were not at risk, which may have contributed to their reluctance to encourage vaccination. This perception is concerning since clinicians sometimes reported using factors such as urban versus suburban residence and socio-economic status to determine a child's risk. While these characteristics are correlated with sexual initiation on a population level, assessing risk on an individual level using this approach is likely to miss at-risk children and may lead to inequalities if certain groups receive stronger vaccine recommendations [6]. Clinicians also differed in their interpretation of the importance of HPV compared with other vaccines, with some asserting that vaccines such as influenza or the meningitis vaccine justified a stronger recommendation than the HPV vaccine. Finally, some clinicians were uncomfortable engaging families about HPV because it is a sexually transmitted infection (STI) and therefore a sensitive topic for families, a finding that confirms prior qualitative research on HPV vaccine communication [33]. In this context, clinicians were hesitant to "push" for vaccination because of concerns about the impact of this approach on their long-term relationship with families.

As these examples reveal, and as others have found,[33,34] clinicians in our sample identified challenges in communicating with ambivalent families and in presenting the scientific evidence for the HPV vaccine. Effective interpersonal and communication skills as well as knowledge of how to educate families, two areas identified as challenges by clinicians in our study, have been recognized as core competencies by the U.S. Academic Council on Graduate Medical Education (ACGME) and prioritized by policymakers focused on improving healthcare quality [35,36]. However, these skills have not historically been emphasized in medical education. Our results highlight the potential benefit of interventions to improve communication between clinicians and parents. Specifically, in the setting of HPV, brief motivational interviews have been suggested as a strategy to engage families who are ambivalent [21]. This technique has been shown to help families identify and clinicians address underlying concerns in a way that allows the clinician to convey respect and empathy while also sharing medical information [37]. Our results suggest that evaluation of this technique in the setting of HPV vaccine decision making is warranted. Additional work is also needed to help clinicians better recognize their own underlying concerns and biases and the ways in which these may impact the presentation and delivery of evidence-based care.

Overall, three prototypes for medical decision making have been defined in the literature: (1) a paternalistic model in which the clinician makes the decision and communicates it to the family, (2) an informed model in which the patient/family gathers information from the clinician and other sources and reaches a decision, and (3) a shared model in which medical and personal information is exchanged and the clinician and family jointly reach a decision [38]. We found that clinicians in our study adopted either the paternalistic or shared model in discussing HPV. Those who used the paternalistic approach believed that they had more success in vaccinating adolescents by presenting HPV as a standard part of the care of early adolescents, without providing additional information about the vaccines. In our sample this appeared to be true, especially among African American patients. However, this qualitative study was not designed to formally test this association. In contrast, those who shared in decision making were less focused on the outcome of vaccine receipt and instead emphasized the importance of involving families in the decision and discussing risks and benefits in detail. Further study is needed to evaluate the impact of both these approaches on decision making, patient satisfaction, and vaccine receipt in diverse primary care populations. Results of this work will help to inform how clinicians should be trained to most effectively address sensitive topics such as HPV vaccination.

Teens in our sample characterized themselves as playing a passive role in vaccine decision making, suggesting that additional strategies for educating and engaging teens about the HPV vaccine as well as other adolescent vaccines may be warranted. Previous studies have found that adolescents are interested in HPV and other STI vaccines;[21] however, this interest may not result in active participation in the primary care setting. Though the American Academy of Pediatrics (AAP) Committee on Bioethics has emphasized the importance of adolescent involvement in decision making,[39] our results suggest that, at least in the short-term, efforts to improve HPV vaccination rates among younger adolescents in the primary care setting may be most effective if targeted primarily at parents. Mothers' approval of vaccination has also been shown to predict vaccine acceptance among older teens,[15] which underscores their central role in decision making.

Teenagers in our study primarily contributed to the vaccine discussion by expressing concerns about pain, a finding supported by other recent qualitative studies [40,41]. Although mothers acknowledged their teens' concerns, the conversation with the clinician typically focused on the mother's distinct concerns. Unlike mothers, teenagers in our study did not appear to associate the HPV vaccine with sex. Other studies have shown that some parents worry that vaccination might encourage sexual activity among teenagers;[42] however, our results should help allay these fears. Consistent with findings that teens had limited knowledge about cervical cancer, Papanicolaou smears,[43] and the HPV virus, [40,41] teens in our study, most of whom were 15 or younger, knew very little about what the HPV vaccine prevented. Among those who had heard of the vaccine, none mentioned sexual transmission. Educating teens about sexuality and sexually transmitted infection prevention has not been shown to promote adolescent sexual behavior,[44] a fact that primary care pediatricians may find helpful to share with parents specifically concerned about the vaccine's association with sex.

This study had several limitations. We did not observe the visits; therefore, our results are based on participant report. Our sample included mostly younger teens, all of whom were accompanied by a parent; they may be more inclined than older or unaccompanied teens to focus on immediate concerns, such as pain, rather than future benefits. We purposively sampled from pediatric primary care practices within one health system and results may not be generalizable to all settings. As a qualitative study, our aim was to generate themes to enhance our understanding of HPV decision making. Nonetheless, we did assemble a sample of families with diverse racial and educational backgrounds and clinicians working in varied practice settings with a wide range of experience, which mitigates the impact of selection bias. Our finding that teens did not appear to associate the HPV vaccine with sex may have been biased by teens' unwillingness to discuss the issue of sex with the interviewers. Additionally, participants were aware that we were studying adolescent vaccination and this could have potentially biased comments. Still, we elicited a broad and diverse set of responses suggesting that participants openly discussed HPV vaccine decision making.

## Conclusions

By exploring adolescent, maternal, and clinician perspectives on HPV vaccine decision making at the point of care, we identified several factors that may help explain low rates of receipt. These include parents' tendency to delay vaccination based on beliefs that their teens were not currently at risk for acquiring HPV and clinician reluctance to engage ambivalent parents about their concerns. We also found that teens were passive participants in vaccine decision making, and that clinicians used two distinct approaches in presenting the vaccine to families. In formulating interventions to improve vaccine delivery in primary care, it will be important to develop strategies to promote effective communication between clinicians and parents at the first opportunity, to evaluate approaches such as motivational interviewing to help clinicians engage reluctant parents in discussion, and to measure the impact of distinct clinician decision making styles on HPV vaccine delivery.

## Abbreviations

(HPV): Human papillomavirus; (CHOP): The Children's Hospital of Philadelphia; (PeRC): Pediatric Research Consortium; (STI): Sexually transmitted infection; (ACGME): Academic Council on Graduate Medical Education; (AAP): American Academy of Pediatrics.

## Competing interests

The authors declare that they have no competing interests.

## Authors' contributions

CCH conducted interviews, participated in data analysis and contributed to drafting the manuscript. ALJ conducted interviews and participated in data analysis. KAF helped conceive of the study, participated in data analysis, and contributed to drafting the manuscript. AGF conceived of the study, participated in data analysis, and contributed to drafting the manuscript. All authors read and approved of the final manuscript.

## Pre-publication history

The pre-publication history for this paper can be accessed here:

http://www.biomedcentral.com/1471-2431/11/74/prepub
